# Extracellular Vesicles: Current Analytical Techniques for Detection and Quantification

**DOI:** 10.3390/biom10060824

**Published:** 2020-05-28

**Authors:** Esther Serrano-Pertierra, Myriam Oliveira-Rodríguez, María Matos, Gemma Gutiérrez, Amanda Moyano, María Salvador, Montserrat Rivas, María Carmen Blanco-López

**Affiliations:** 1 Department of Physical and Analytical Chemistry, University of Oviedo, 33006 Oviedo, Spain; serranoesther@uniovi.es (E.S.-P.); oliveiramyriam@outlook.es (M.O.-R.); moyanoamanda@uniovi.es (A.M.); 2 Instituto Universitario de Biotecnología de Asturias, University of Oviedo, 33006 Oviedo, Spain; matosmaria@uniovi.es (M.M.); gutierrezgemma@uniovi.es (G.G.); 3 Department of Chemical and Enviromental Engineering, University of Oviedo, 33006 Oviedo, Spain; 4 Department of Physics & IUTA, University of Oviedo, Campus de Viesques, 33204 Gijón, Spain; salvadormaria@uniovi.es (M.S.); rivas@uniovi.es (M.R.)

**Keywords:** extracellular vesicles, characterization, quantification, ELISA, lateral flow immunoassay, nanoparticle tracking analysis, asymmetrical-flow field-flow fractionation

## Abstract

Since their first observation, understanding the biology of extracellular vesicles (EV) has been an important and challenging field of study. They play a key role in the intercellular communication and are involved in important physiological and pathological functions. Therefore, EV are considered as potential biomarkers for diagnosis, prognosis, and monitoring the response to treatment in some diseases. In addition, due to their properties, EV may be used for therapeutic purposes. In the study of EV, three major points have to be addressed: 1. How to isolate EV from cell culture supernatant/biological fluids, 2. how to detect them, and 3. how to characterize and quantify. In this review, we focus on the last two questions and provide the main analytical techniques up-to-date for detection and profiling of EV. We critically analyze the advantages and disadvantages of each one, aimed to be of relevance for all researchers working on EV biology and their potential applications.

## 1. Introduction

Understanding the biology of extracellular vesicles (EV) has been an important and challenging field of study since their first observation in 1946 [1]. These small membrane-bound particles are produced by several mechanisms: By fusion of multivesicular bodies and the plasma membrane (usually referred as exosomes), or directly by plasma membrane budding in response to intracellular or extracellular stimuli [2,3,4]. In the current article, the term “extracellular vesicle” will preferably be used, according to the International Society of Extracellular vesicles (ISEV) recommendation [5] to refer to “particles naturally released from the cell that are delimited by a lipid bilayer and cannot replicate”.

EV play a key role in intercellular communication since they contain lipids, proteins, and nucleic acids, which can be transferred to the recipient cells. Therefore, they are involved in important physiological and pathological functions and are considered as potential biomarkers for diagnosis, prognosis, and even for monitoring the response to treatment in some diseases [6,7]. In addition, due to their properties, EV can be potentially used for therapeutic purposes.

A large variety of methods have been developed and implemented to allow EV isolation and enrichment. Differential centrifugation at increasing speeds is still by far the most common. Immunoaffinity capture-based methods enable the selection of EV derived from specific cell types, whereas size-exclusion chromatography is used to separate exosomes from other EV with different sizes. Precipitation reagents may also be used to isolate EV in shorter times and lower centrifugation speeds. See references [8,9] for critical reviews on current methods for EV isolation.

In order to gain insight into EV biology, a proper characterization is required upon isolation. Quantification of EV remains a challenge, although a number of techniques are currently available to the researcher to characterize and quantify these vesicles. Thus far, none of these techniques enables a full characterization, due to their heterogeneity in terms of size and the intrinsic complexity of biological samples. Therefore, the strengths and weaknesses of each technique must be taken into account when performing the detection and profiling of EV. We provide in this article a SWOT analysis (Strengths–Weaknesses–Opportunities–Threats) of the most commonly used methods to detect and quantify EV. The principles of these methods are explained and works using these techniques for EV studies are provided. In addition, we outline other emerging techniques with high potential for the study of EV.

## 2. Enzyme-Linked Immunosorbent Assay (ELISA)

Enzyme-linked immunosorbent assay is a commonly used method for detection and quantification of an analyte (e.g., peptides, proteins, or antibodies). The basis of this technique is the antigen–antibody (Ag–Ab) reaction. It is a reversible chemical reaction, where the binding must overcome the repulsion forces between both molecules. There are several weak and non-covalent interactions involved in the antigen–antibody complex formation, such as van der Waals forces, hydrogen bonds, electrostatic, and hydrophobic interactions [10].

As previously mentioned, an antibody is used to detect the target molecule. This antibody is linked to an enzyme, which eventually produces a detectable signal (most commonly a color change) after addition of a substrate. The measurement of this signal, together with an appropriate standard, allows the quantification of the levels of the substance under study.

Based on this simple procedure, there are many modifications that can be performed in an ELISA assay to optimize the detection of the target analyte. ELISAs are usually performed in 96-well polystyrene plates, which enable not only the immobilization of antibodies and proteins, but also the analysis of multiple samples per assay. Thus, this method offers a high flexibility regarding its design and procedure. A summary of the basic steps in ELISA assays could be as follows:

- Coating of the plate wells with an antigen or an antibody (capture).
- Addition of the standard points and the samples to the wells.
- Binding of the antigen to the antibody (or of the antibody to the antigen) coated on the plate.
- Washing of the wells to remove any non-specific bound materials.
- Addition of a primary antibody (detection).
- Addition of a secondary antibody conjugated to an enzyme.
- Addition of the substrate.
- Measurement of the color reaction.

### 2.1. Types of ELISA

**Figure 1:**
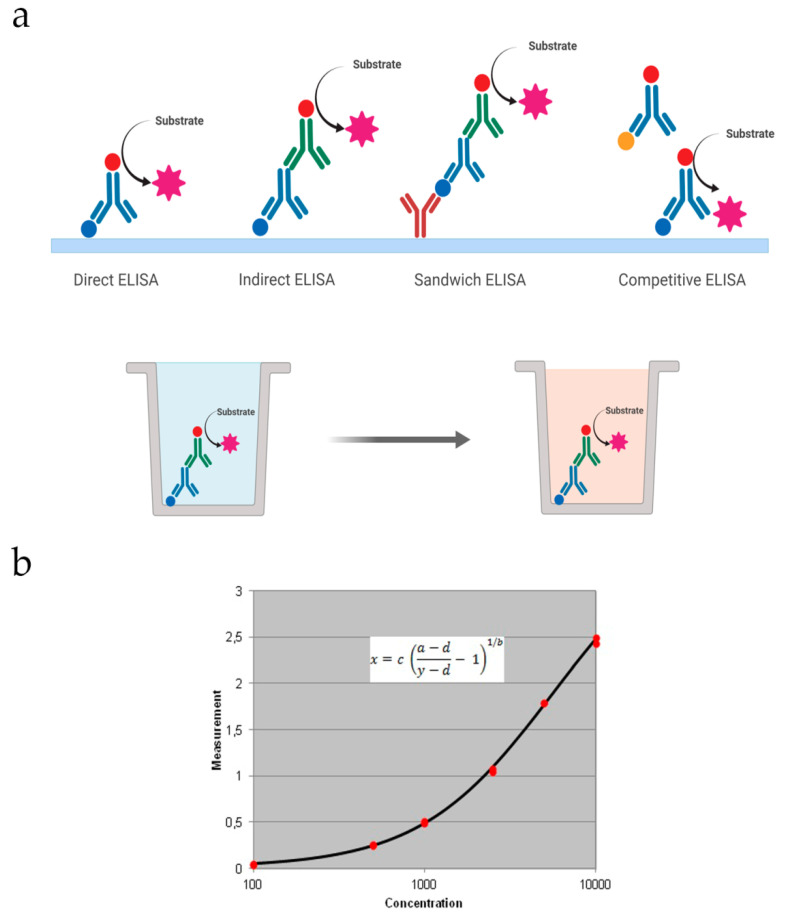
(**a**) Schematic representation of the various types of ELISA that can be performed on a plate; (**b**) an example of a standard curve employed to quantify the concentration of an analyte in the ELISA assay, in this case using a four parameter logistic regression.

There are four basic formats that can be further improved or optimized based on the substance we want to detect (Figure 1a).

- Direct: The target antigen is immobilized on the well and detected with an antibody conjugated to an enzyme. Then the substrate is added for the colored reaction. This makes this type of ELISA faster than the others.
- Indirect: In this case, a specific primary antibody is used to detect the target antigen, which is immobilized on the well. Then, a secondary antibody coupled to an enzyme is added to recognize the primary antibody. It has greater sensitivity, since the primary antibody contains different epitopes that can be bound to the secondary antibody.
- Sandwich: In this assay the capture antibody is coated on the well and the sample is added. It can be direct or indirect based on the detection antibody. If it is conjugated to an enzyme, detection is direct. If it is unlabeled, a secondary antibody conjugated will be used for indirect detection.
- Competitive: The primary antibody is incubated with the sample antigen. The Ag–Ab complexes are added to a plate coated with the same antigen and unbound antibodies are washed off. There will be a competitive reaction between the sample antigen and the antigen bound to the plate. Therefore, the more antigen in the sample, the lesser primary antibody available to bind the antigen coated.

Data obtained from ELISA assays are commonly represented in a graph with the signal (colorimetric or fluorometric) vs. known concentrations of the target antigen that will be used to produce a standard curve (Figure 1b). Then this data is used to measure the concentration of the unknown samples directly on the graph or by using curve fitting software.

### 2.2. ELISA for Detection of Extracellular Vesicles

The increasing interest in extracellular vesicles has led researchers to find appropriate methods to quantify them. In this sense, ELISA is a powerful tool, since the use of tetraspanins offers high specificity for the detection and measurement of specific subsets of EV, such as exosomes. Transmission electron microscopy (TEM) could also be used to determine their size and to estimate the quantity of EV. However, with the appropriate standard points, ELISA is preferable. Although the total concentration of the EV cannot be inferred due to the heterogeneity and subtypes of EV with different membrane proteins that can be used as markers, this method can detect and quantify EV from a specific cellular origin. Exosomes derived from ovarian cancer cells can be identified by their expression of CD24 and EpCAM [11], showing the potential role of EV in cancer diagnostics.

### 2.3. SWOT Analysis of ELISA

#### 2.3.1. Strengths and Weaknesses

As previously mentioned, ELISA techniques can be modified for optimization according to the analyte of interest. Its flexibility allows a broad range of possibilities to analyze EV with in-house designed ELISA.

ELISA assays for most of proteins of interest are commercially developed. Currently, there are ELISA kits available for detection and quantification of EV, although they may not be the optimal choice under some conditions [12]. In-house ELISA assays have also been developed for exosome detection and quantification [13,14].

However, this method is a multi-step assay with multiple wash steps too, where more errors can occur. It is time consuming and the variability inter-assays and intra-assays must be taken into account. It requires low sample volumes and offers both qualitative and quantitative results. In addition, multiple samples can be performed in a single assay.

#### 2.3.2. Opportunities and Threats

The flexibility of the ELISA method for optimization enables its application for detection of EV derived from different sources (cell culture and biological fluids, such as plasma, serum, urine, and cerebrospinal fluid), which has potential interest in biomedical research. With a sufficient volume, the same sample can be analyzed several times to study different targets of interest. In addition, the time-consuming process may be overcome in paper-based ELISA assays [15] or by pre-enrichment of the sample and subsequent detection using nanocubes with enzyme-like characteristics [16]. The development of reference materials may enhance ELISA-based approaches, since they may be designed according to the cell origin, surface density of the antigen, etc. [17]. This method is affordable for laboratories and research groups. Moreover automation of ELISA is available, improving sensitivity and reproducibility and reducing the variability.

The ELISA principle may be transferred to a microfluidic device to obtain lower detection limits [18]. Development of biosensors may also displace the traditional ELISA method, offering quicker assay times and the possibility to design devices for specific markers. Also, the development of multiple-targeted detection systems would significantly reduce the time and the costs of analysis, avoiding the performance of different ELISA assays for the same sample.

## 3. Lateral Flow Immunoassay Systems

**Figure 2:**
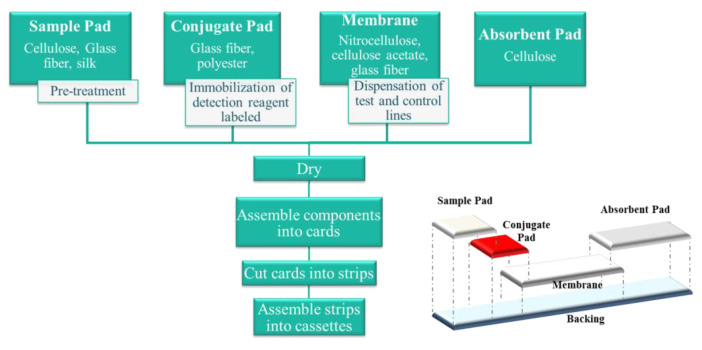
Outline of a lateral flow immunoassay manufacturing process. The materials and process typically required are detailed in the diagram. The bottom figure represents the assembling process.

The lateral-flow immunoassay (LFIA) is a well-established point-of-care diagnostic technology which offers the advantages of widening accessibility to diagnosis, reduced costs, rapid analysis times and being user-friendly [19]. The basis of LFIAs is based on the same logic as ELISAs, where the capture antibody or immobilized antigen is bound to a membrane, usually nitrocellulose, instead of a plastic well. Unlike ELISAs, the advantage of LFIAs is that the entire assay can be performed in a single step and in a few minutes, avoiding the long incubation times and tedious steps involved at the multiple-step ELISA [20]. The design of the LFIA consists of integration of various components (sample pad, conjugate pad, membrane, and absorbent pad) in a serial, lateral manner with a small overlap connecting each of the parts, which allows the sample flow (Figure 2).

When the test is carried out, a small sample volume is applied to the sample pad. The sample migrates to the conjugate pad, where the detection reagent (label) conjugated to a specific biological component has been immobilized. The analyte interacts with the conjugate and both migrate into the membrane, where the capture reagent, other specific biological component, has been immobilized. Excess of sample and reagents continue to flow to the absorbent pad, which serves as storage and waste [21]. Of all of the components, the membrane is probably the most important part of the test platform. The overall yield is influenced by far by its physical morphology and chemical composition (related with protein interactions). The speed with which the complex of the sample analyte and the label is transported through the membrane determines the reaction time, and therefore the assay sensitivity [22].

In addition to the materials and manufacturing process of the strips, other key elements of a highly performing LFIA system are as follows: Assay format, biological recognition reagents, signal generation technologies (labels), and interpretation and signal transduction technologies.

### 3.1. Lateral Flow Immunoassay Formats

**Figure 3:**
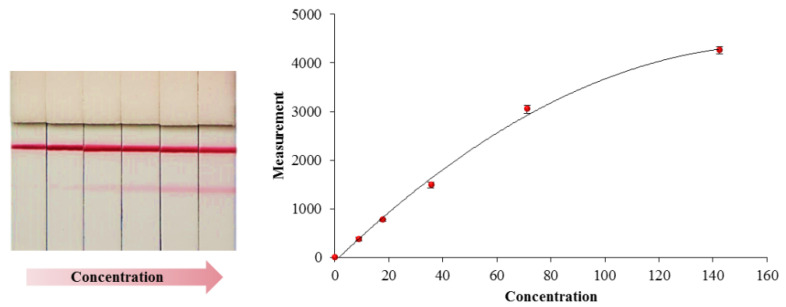
An example of a standard curve employed to quantify the concentration of an analyte in the lateral-flow immunoassay (LFIA) (Adapted from [23]).

There are various formats for LFIAs, being the sandwich approach by far the most common. A typical sandwich LFIA is the pregnant test, where the presence of analyte is evidenced by the appearance of two lines in the detection zone, the test and control lines. This test line is the result of a complex formed between the biological reagent immobilized in the membrane and the target analyte already bound with the labeled antibody pre-loaded on the conjugate pad. The higher the analyte concentration, the greater the signal intensity. A typical response obtained using this approach can be observed in Figure 3. This approach cannot be employed with low molecular weight analytes, usually haptens, for which only one antibody may bind to it. In this case, a competitive format is possible, where a positive result is indicated by the absence of the test line. Many drugs of abuse assays utilize this format [21].

Within the design of the LFIA, another possibility is the use of a multiplex detection format. Such format is applied for detection of more than one target and assay is usually performed over the same strip, containing test lines equal to number of analytes to be analyzed, which implies the immobilization of different capture reagents [24].

#### 3.1.1. Biological Recognition Reagents in Lateral Flow Immunoassays

There are many different biological recognition elements that can be employed in the design of a LFIA, but antibodies are by far the most established and commonly used. It is very important to carry out a careful analysis and selection of them, since the performance of the assay will be affected by the class of antibodies, their characteristics (including specificity and affinity) and also by the association rate constant. It should be noted that LFIAs are particularly demanding in terms of the affinity required in the interactions between the antigen and the antibody due to the short-assay time [25].

#### 3.1.2. Signal Generation and Interpretation Technologies

The choice of a label in a LFIA development process normally depends on requirements of sensitivity, reproducibility, stability, ease of conjugation with biological reagents, complexity (the need for readers), and cost. For low-cost qualitative applications, optical methods are the most explored because their simplicity (naked-eye detection of the presence or absence of the test line) and quickness [21]. A wide variety of nanomaterials can be used as colorimetric labels in LFIA, such as latex beads, gold nanoparticles (AuNPs), quantum dots (QDs), magnetic beads (MB), liposomes, and enzymes. The development of quantitative assays requires additional readers which allow measurement the signal produced at the test line of the strip. Although optical readers are still the preferred (scanners or automated strip readers), there is growing use of fluorescent, chemiluminescent, electrochemical, and magnetic measurement systems [26].

### 3.2. Lateral Flow Immunoassay for EV Detection

As previously discussed for ELISA, the protein content in the EV surface allows the detection of theses vesicles by LFIA. Tetraspanins are especially enriched in the membrane of exosomes, and they are often used as exosome biomarkers due to their high abundance in virtually any cell type [27]. Using combinations of antibodies against these proteins, it is possible to develop LFIAs for the detection of exosomes from different sources [14,28]. Given the small size of exosomes, they can flow through the membrane without blocking the pores or obstructing the flow, until they are captured by the antibody immobilized on the detection line. Moreover, the LFIA platform can be further developed into a multiple-target assay by using different antibodies on different test lines. This allows the detection of a broad range of EVs based on their surface protein content [29]. A calibration curve may be used to quantify EV, as in ELISA. Additionally, this LFIA test could be used as an auxiliary method in the EV isolation process, for example, to rapidly determine the fraction with the highest EV concentration in size exclusion chromatography.

### 3.3. SWOT Analysis of LFIA

#### 3.3.1. Strengths and Weaknesses

As previously mentioned, LFIA techniques can be modified for optimization according to the analyte of interest. Its flexibility allows a broad range of possibilities to analyze EV without the need for significant or complex infrastructure. These platforms are ease to use, in a one-step assay, with minimal operator handling. Thus, LFIA is a great tool for cost-effective on-site detection. Since the microRNA (miRNA) expression in EV may be altered in some diseases [30,31], the development of nucleic acid lateral flow systems that enable miRNA detection [32,33] confirms the potential of this technique.

Although LFIA allows quick and simple point-of-care diagnosis, their simplicity can limit their performance and in many cases more complex devices are necessary for accurate diagnosis. A limitation commonly reported is their lack of sensitivity, which can be improved by the use of different labels or signal enhancement strategies [34,35]. In addition, often visual colorimetric measurements by naked-eye are not sufficient due to the variation in visual perception or when a more accurate result is required. For this reason, in order to achieve quantitative analysis, LFIA platforms often need to be coupled to an appropriate detection reader.

#### 3.3.2. Opportunities and Threats

The flexibility in the design of LFIA platforms allows that a simple change of antibodies can lead to a more specific system, not only for the detection of other populations of EVs, but also to investigate or to detect a concrete disease marker. For applications using blood or other complex matrixes, special sample pad materials are available which filter cells or big particles from obstructing the membrane without the need of sample pretreatments. On the other hand, the increase in the usage of mobile phones converts them in potential tools, as the phone camera can be used as a portable detector for digitizing, storing and transferring the results of LFIA in the on-field and off-lab applications.

In addition to the lack of sensitivity previously commented, extreme test conditions (temperature, humidity) can affect the performance of these devices, causing negative effects on the reagents used, modifying the rate of liquid migration or the recognition between molecules. These deficiencies can lead in the development and use of more sophisticated and robust systems, replacing the LFIA use. In the case of EV, the differences in protein composition, localization, and density of tetraspanins at the exosome surface can markedly influence their detection in LFIA. Therefore, the development of specialized LFIAs for specific exosome subpopulations is necessary.

## 4. Nanoparticle Tracking Analysis (NTA)

Nanoparticle tracking analysis (NTA) is an increasingly popular technique to determine particle size distribution and concentration. It is based on the individual detection of particles and the tracking of their Brownian displacements by recording the scattered light of a laser beam with a charge-coupled device (CCD) camera.

The software relates the speed of motion to the diffusion constant *D*, which in turn is related to the diameter of an equivalent spherical particle (hydrodynamic diameter *d*) by the Stokes–Einstein equation:

(1) $$D=\frac{Tk_{B}}{3\pi \eta d}$$

where $k_{B}$ is Boltzman’s constant, T is the temperature, and *η* the viscosity of the solvent.

To take into account the fact that NTA tracks Brownian motion in two dimensions, a variation of the Stokes–Einstein equation is used to determine the diffusion coefficient by measuring the mean squared displacement:

(2) $$\bar{{\left(x,y\right)}^{2}}=\frac{4Tk_{B}t}{3\pi \eta d}$$

where *t* stands for the sampling time.

NTA can detect and characterize particles in real time ranging from 10–20 nm to 1000–2000 nm, approximately. The amount of light scattered by the particles and the optics of the equipment are the main factors that determine the lower limit of detection. NTA is suitable for both monodisperse and polydisperse samples. Therefore, different EV subtypes may be characterized using this system. Furthermore NTA provides additional information by measuring other parameters such as zeta potential, the relative intensity of light scattered, or particle concentration [36]. Since this technique individually tracks and sizes each particle, a low concentration of particles is required to achieve accurate results (usually 10^6^–10^10^ particles/mL). In addition, NTA enables the visualization of the particles and the detection of specific vesicles by fluorescent labeling [37,38]. This is also an advantage in comparison with conventional flow cytometry methods, which only detects particles above 200–300 nm so EV cannot be analyzed directly and requires further handling for its analysis.

### 4.1. NTA for Analysis of EV

NTA is one of the most widely used methods to study size distribution and concentration of EV, and it has potential application in the field of biomedicine. Size distribution of EV may differ in pathological conditions [39]. In fact, higher amount of EV were found in patients with breast cancer [40], or with prostate cancer [41] in comparison with healthy controls using the NTA system. The number of circulating EV was found elevated in patients with chronic fatigue syndrome (CFS), which was determined by NTA [28]. Recently, EV numbers were too found higher in a rat model of Parkinson disease [42]. Thus, the use of this technique is making significant progress on the study of EV under pathological conditions.

**Figure 4:**
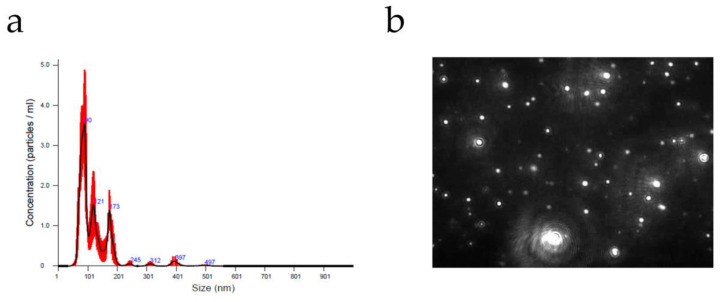
(**a**) Size distribution and concentration of E extracellular vesicles (EV) isolated from serum with the corresponding nanoparticle tracking analysis (NTA) video frame (**b**). NTA measurements were performed using a NanoSight LM10 instrument (Malvern, Worcestershire, UK) at Nanovex Biotechnologies S.L. (Llanera, Spain).

A representative graph than can be obtained in a NTA analysis is shown in Figure 4.

### 4.2. SWOT Analysis of NTA

#### 4.2.1. Strengths and Weaknesses

With this technique, it is feasible to measure and quantify EV at once. The information provided by NTA it is of great interest in several fields, including biomedicine. For polydisperse samples different subtypes of EV can be distinguished in the same sample. In addition, the required sample volume and preparation is minimal compared to more sophisticated methods as electron or atomic force microscopy. However, unlike dynamic light scattering system (DLS), this equipment requires a specialized/skilled operator. The optimization of the settings for the analysis of particular types of EV samples may require time. In addition, the use of this equipment may not be affordable or available for all research groups.

It is particularly important in the case of EV to take into account that there are several factors that may produce an overestimation of the average size with NTA. One of the main reasons is its reduced sensitivity for the smaller sizes (<50 nm), which produces a shift of the center of the distribution to larger sizes. Secondly, as any light scattering-based technique, NTA measures the hydrodynamic size which corresponds not only to the solid phase but also to the electric dipoles of the solvent that adhere to it; it is well known that the difference between hydrodynamic size and, for instance, TEM size, is larger for small particles (approximately 50% for sizes ca. 15 nm and only 5% for 100 nm) [43,44], which again possibly implies average size overestimation. In the third place, the length of the trajectories measured is different for the different sizes. Finally, agglomeration, inhomogeneous distributions, and interparticles interactions can produce the violation of the Stokes–Einstein diffusion. Some of these size effects can be remedied by a careful choice of the protocol of data processing (see Kestens et al. [45] and references therein).

#### 4.2.2. Opportunities and Threats

As mentioned above, NTA is the most widely used method for EV characterization and concentration. Phenotyping of particular types of EV may be performed by labeling fluorophore-conjugated antibodies, which is not feasible with conventional flow cytometry. One of its potential applications is biopharmaceutical development in drug delivery systems, where their characterization to ensure quality and stability is a key factor.

Despite all these advantages, current methods of EV isolation cannot separate EV according to their size. NTA allows size characterization but not their separation. In this sense, asymmetrical-flow field-flow fractionation (AF4) is a technique which enables EV fractionation according to their size. This technique is relatively new in the field of EV study, yet it requires further work on standardization for their analysis to achieve reproducible results [46,47].

## 5. Asymmetrical-Flow Field-Flow Fractionation

The asymmetrical-flow field-flow fractionation (AF4) is a technique that enables the separation of particles or molecules according to their diffusion coefficient. It was developed in the early 1980s based on the field-flow fractionation (FFF) system, which separates the particles in a laminar velocity gradient [48,49].

AF4 can fractionate and characterize macromolecules (e.g., proteins) and nano- to micron-sized particles (e.g., organelles and cells) in a very wide size range (approximately 2 nm to >1 mm) [50,51]. Although the method can be considered chromatography-like it does not rely on a stationary phase and, thus, shear forces during separation are small. Furthermore, pre-injection filtering of samples is often not required [51].

There are three basic steps in an AF4 experiment:

- Sample injection
- Sample focusing
- Fractionation

**Figure 5:**
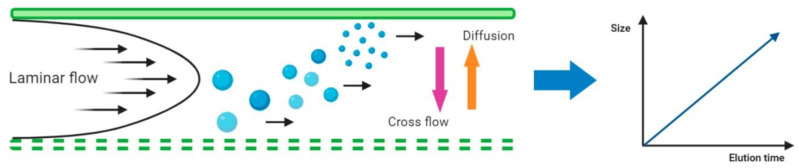
Asymmetrical-flow field-flow fractionation (AF4) channel cross section showing the separation principle (adapted from [52]). Elution occurs by means of a laminar flow in parabolic pattern. A cross flow drives the particles towards the membrane, and is counteracted by the size-related diffusion properties of the particles.

In the AF4 system a laminar flow carries the sample through the chamber, where particles will be separated. Then a cross-flow is applied perpendicularly to this chamber, which consists of an upper plate (impermeable) and a bottom permeable plate (ultrafiltration membrane with a certain pore size) separated by a spacer. The cross-flow drives the different components of the sample towards the ultrafiltration membrane of the channel, the accumulation wall, where they are confined to a thin concentrated layer. The field induced transport is counteracted by a diffusional transport, giving rise to a steady-state concentration distribution. Hence the application of this flow vertically distributes the particles in the sample, and their respective diffusion coefficients will determine the proximity to the ultrafiltration membrane of the channel. Since smaller particles have higher Brownian motion they migrate further and faster than larger particles or molecules into the flow. Therefore, components with smaller sizes will elute first so the elution time will correspond to the size of the particle. A schematic representation of the AF4 system is shown in Figure 5.

It is important to do a proper selection of the membrane material regarding the minimization of unwanted interactions between the sample components and the ultrafiltration membrane since sample components could be adsorbed onto the membrane surface. Repulsive interaction may also have to be considered as it can interfere with the fractionation. Proteins are more likely to sometimes require optimization in the aspect of adsorption as they are inherently surface active [51]. Aqueous carrier liquids whose composition varies depending on the analyte are commonly used in AF4 although other solvents may be utilized. However, pure water is not advisable since any electrostatic interaction acting during separation may cause disturbances in the elution of sample components and poor reproducibility. Moreover, optimization of the amount injected onto the channel is necessary to eliminate or minimize overloading. This is especially important if accurate data, such as diffusion coefficients, are to be obtained from elution times. The optimization is straightforwardly performed by injecting various amounts of sample for which the retention time should be constant if no overloading occurs [51].

The AF4 system may be coupled to a multi-detection system, such as dRI (differential refractive index), flow DLS (dynamic light scattering), or MALS (multi-angle light scattering) for further characterization of the particles. Therefore a combination of AF4 and these detection systems can provide information on the root mean square (rms) radius, the hydrodynamic size or the molar mass of the separated particles, respectively.

### 5.1. AF4 for Analysis of Extracellular Vesicles

AF4 is currently a state-of-the art method for the separation of particles and macromolecules, showing its utility for multiple applications [53]. Since EV that can be isolated from complex samples are heterogeneous, this method has potential application in the separation of EV subpopulations, which is not feasible with the methods of EV purification currently available. Furthermore, their elution may enable further analyses of the collected fractions, such as mass-spectrometry analysis [50].

Nevertheless, there are still few works reporting EV fractionation from biological samples using AF4 system. Vesicles from the immortalized human mesenchymal stem cell line B10 [54] and from immortalized human neural stem cells (HB1.F3) have been separated using AF4, allowing the fractionation of exosomes according to differences in hydrodynamic diameter ranging from 30 nm to 100 nm [50], which are the smallest EV. To our knowledge it was the first study providing evidence of nanometer-scale, size based fractionation of exosomes with morphological confirmation and proteomic analysis [50]. In addition, size fractionation of exosomes derived from HB1.F3 cell line was coupled to subsequent in-solution digestion and proteomic analysis by using nanoflow liquid chromatography/electrospray ionization-tandem mass spectrometry (nanoflow LCESI-MS-MS), and were analyzed by transmission electron microscopy (TEM) to morphologically confirm their identification as exosomes [50]. Thus, without a large scale culture of these cells, a large amount of valuable information was obtained. The use of AF4 coupled to different detectors has been used to characterize exosomes derived from a mouse melanoma cell line (B16-F10), showing high reproducibility between samples [55]; similar results in terms of reproducibility have been reporting in the study of urine-derived EV [56]. Other work also distinguished EV subpopulations when analyzing a standard of exosomes purified from a B cell line, which is commercially available [57]. More recently, Zhang et al. [58] established AF4 protocols to characterize small EV from different cell lines, even describing a subpopulation of non-membranous particles (exomeres).

**Figure 6:**
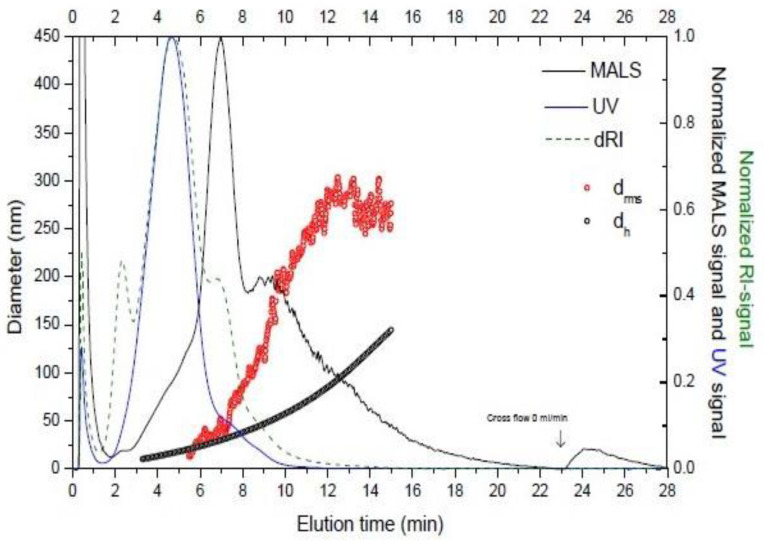
Elution profile of an EV sample. The graph shows the hydrodynamic diameter (dh, black open circles), the diameter of gyration (drms, red open circles). Signals are dRI (differential refractive index) (blue line), MALS (multi-angle light scattering) (black line), and UV signal (green dashed). The analysis was carried out at Solve Research Consultancy AB (Lund, Sweden) using an AF4 instrument (Eclipse 2 Separation System, Wyatt Technology Europe, Dernbach, Germany), which was connected to a MALS detector (Dawn Heleos II, Wyatt Technology, Dernbach, Germany) operating at a wavelength of 664 nm, a RI detector (Optilab T-Rex, Wyatt Technology, Dernbach, Germany)) operating at 658 nm, and an UV light detector (Hewllet Packard 1100 Series G1315A, Agilent Technologies, Kista, Sweden) operating at 240 and 280 nm wavelength.

All the works cited above emphasized the need to set the experimental parameters to optimize the fractionation of extracellular vesicles and the most recently described exomeres [59]. Thus, factors like channel thickness, pore size of the membrane, flow rates, and elution times significantly influence the procedure and must be adapted to each type of sample analyzed. Figure 6 shows an example of a fractogram obtained of EV isolated from a biological sample.

### 5.2. SWOT Analysis of AF4

#### 5.2.1. Strengths and Weaknesses

The AF4 system has been shown to be one of the most effective methods for size fractionation of EV and to be highly reproducible [57]. Moreover, the combination of AF4 with multiple detectors provides a great amount of information about the vesicles. However, the use of this technology requires a skilled operator and a thorough work to find the optimal settings for EV fractionation. More reports are needed in this field to gain insight into the benefits of this technology.

#### 5.2.2. Opportunities and Threats

Further development of this system may allow large scale fractionation of EV, which may have potential clinical and pharmaceutical applications. Eluted fractions can be further analyzed by TEM and mass spectrometry, but it is still necessary to investigate whether they can be employed for functional in vivo or in vitro studies.

## 6. Other Emerging Techniques

In the last years a substantive progress has been made in the design of new analytical platforms which can rapidly detect, characterize, and quantify EV. Specifically, novel miniaturized systems which enable a rapid sample preparation and analysis. These platforms will help to overcome the current extensive processing of samples to obtain EV, and therefore will enhance their clinical potential use as biomarkers or as therapeutic drug carriers.

### 6.1. Conventional and High Resolution Flow Cytometry

Flow cytometry is a technique for quantitative single cell analysis. Cells in suspension flow in single file, surrounded by a narrow fluid stream. Once they pass through a focus laser beam, cells and fluorescently labeled cell components are excited and emit light. These signals are then detected by a series of photodiodes and the data are analyzed through a computer system. Its robust statistical power and the ability to analyze multiple parameters make it a useful technique to analyze and characterize EV. However, due to their small size and low refractive index, conventional flow cytometers may not properly detect single vesicles smaller than 500 nm [60,61]. The use of microbeads to attach them to the EV can overcome this limitation and semi-quantitative assays can be performed. Hence, EV can be studied by conventional flow cytometry to gain insight into their origin, function and/or clinical significance [62,63,64,65,66]. Recently, high-resolution flow cytometers have been developed to specifically address the analysis of different populations of EV [67,68,69]. As in the case of AF4, standardized procedures for both conventional and high-resolution flow cytometry are required for measurement of EV number, size, and their phenotyping [70,71].

### 6.2. Tunable Resistive Pulse Sensing (tRPS)

**Figure 7:**
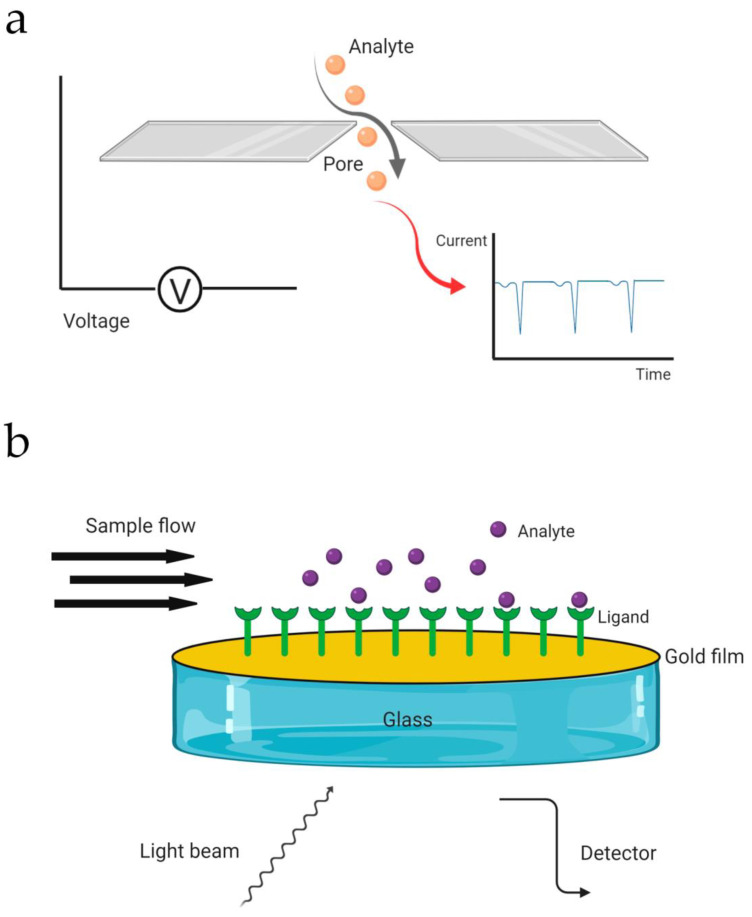
Simplified schematic representation of (**a**) tunable resistive pulse sensing (tRPS) and (**b**) surface plasmon resonance (SPR).

This method is a technology that, like NTA, determines particle concentration and size characterization of EV. It is based on a membrane with nanosize pores, which separates two fluid cells. A voltage is applied to the nanopore; as particles move through the nanopore the current is disrupted, and the appearance of “peaks” or “pulses” is observed (Figure 7a). These pulses are proportional to the volume of particles, and their flow rate is proportionate to the concentration. Since EVs are heterogeneous in terms of size, characterization using tRPS is challenging. In this sense, the work of Coumans et al. [72] provided a thoughtful study in order to gain reproducibility and optimize the settings when measuring EV size and concentration. In addition, it has been reported that biological fluids can be directly employed on this platform, without the need of prior EV isolation [73].

### 6.3. Surface Plasmon Resonance (SPR)

Surface plasmon resonance is a phenomenon that occurs when a light is incident upon a metal film, at the interface between media with different refractive indices, through a glass prism. The refractive index at the prism side does not change, whereas this index at the metal surface side will change when traces of mass (e.g., proteins or vesicles) are brought onto the metal film. The subsequent reflection is detected, and the SPR response can be utilized to determine the adsorption kinetics of the analyte (Figure 7b). The main advantage of this technique is that it enables a real-time measurement of the analyte. For it can be utilized to study binding events within 200 nm from the metal film, SPR has been used for detection, profiling, and particle concentration of a subpopulation of EV, exosomes [74,75]. Furthermore, using the phenomenon of the SPR, a colorimetric nanoplasmonic assay was recently developed to determine EV concentration [76].

### 6.4. Sensors Based on Microfluidics and/or Magnetic Particles

Microfluidics technologies enable detection and analysis of small volume of samples, and are promising in the field of isolation of EV. In addition, microfluidics can be combined with other techniques in order to quantify EV. The magnetic behavior and the physical properties of magnetic particles provide widespread applications in a variety of fields of expertise, including biotechnology, biomedicine, and engineering. As with microfluidics, the use of magnetic particles is emerging not only for isolation of EV, but also for their detection and quantification combined in portable systems.

The work of Rho et al. [77] shows the development of a platform using microfluidics for a first step of enrichment of red cell-derived microvesicles, coupled to a magnetic sensor for detection and quantification. In line with this, a microfluidic device with pairs of electrodes was designed in order to detect and quantify exosomes using alternating current electrohydrodynamic methodology [78]. Circulating exosomes from plasma of ovarian cancer patients were enriched using magnetic microbeads in a microfluidic device for further detection and quantification using different exosomal tumor markers [79]. More recently, an interesting approach using microfluidics was proposed by Liu et al. [80]. Single exosomes immunocomplexes were immobilized on magnetic microbeads, which were further encapsulated into droplets. Then the droplets are detected in the droplet microfluidic system, achieving a limit of detection of 10 exosomes/µL. Therefore, the employment of microfluidic-based technologies is a promising technique in the EV-related research, with potential clinical applications.

The work of Jeong et al. [81] reports the development of a sensor which combines the use of magnetic particles to capture extracellular vesicles with electrochemical detection, and eventually determination of the concentration showing good reliability. Another approach using magnetic nanoparticles is proposed by He et al. [82]. Magnetic beads are used to capture extracellular vesicles; these complexes are further anchored to copper oxide (CuO) nanoparticles, and unbound particles are removed. Cu ions (Cu^2+^) are obtained and then reduced to fluorescent copper nanoparticles (CuNP). The fluorescence emitted is eventually used to estimate the concentration of extracellular vesicles.

## 7. Conclusions

The heterogeneity of EV in terms of size, origin, and composition has made challenging their study. Up to date a variety of methods are available for EV isolation, although proper standardized protocols are still required. Detection and characterization are as challenging as the isolation step. Different technologies have been developed in order to characterize and quantify EV. We have summarized these techniques, their basis, their application in EV research, and a critical review of the main advantages and drawbacks in a SWOT analysis (Table 1). Since other emerging techniques have arisen and may potentially be used to quantify EV in a more accurate and specific manner, we provide an overview on the latest technologies that are currently been used for EV detection and quantification.

## Figures and Tables

**Table 1:** Summary of the SWOT (Strengths–Weaknesses–Opportunities–Threats) Analysis of Different Techniques for EV Detection and Quantification.

	Strengths	Weaknesses	Opportunities	Threats
**ELISA**	Flexibility Commercial kits	Multistep Time consuming	Detection of EV from different sources Affordable Automation available	Development of biosensors Multiple-targeted detection systems
**LFIA**	Cost-effective on-site detection	Lack of sensitivity	Potential portable detectors	Development of more robust systems
**NTA**	Measurement and quantification	Not affordable/available for research groups	Phenotyping of EV sub-populations Applications in drug delivery systems	AF4 enables EV fractionation
**AF4**	Size fractionation High reproducibility	Requires skilled operator	Potential clinical and pharmaceutical applications	Eluted fractions may not be used for further functional studies
